# Prevalence of human anelloviruses in Romanian healthy subjects and patients with common pathologies

**DOI:** 10.1186/s12879-018-3248-9

**Published:** 2018-07-17

**Authors:** Sonia Spandole-Dinu, Dănuț Gheorghe Cimponeriu, Anne-Marie Crăciun, Irina Radu, Silvia Nica, Mihai Toma, Oana Andrada Alexiu, Corneliu Sorin Iorga, Lavinia-Mariana Berca, Remus Nica

**Affiliations:** 1 Department of Genetics, University of Bucharest, Bucharest, Romania; 2 Nutrition and Metabolic Diseases Dr. N. Paulescu, National Institute of Diabetes, Bucharest, Romania; 3 Bucharest Emergency University Hospital, Bucharest, Romania; 4 Dr. Carol Davila Central Military Emergency University Hospital, Bucharest, Romania; 5 National Institute of Research and Development for Food Bioresources, 6 Dinu Vintila, 021102 Bucharest, Romania

**Keywords:** TTV, TTMDV, TTMV, Diabetes, Breast cancer

## Abstract

**Background:**

Human anelloviruses (TTV, TTMDV and TTMV) are at high prevalence all across the globe, having also a controversial disease-inducing potential. This study aimed to estimate the prevalence of anelloviral DNA in the Romanian human population and to investigate the association of infections with common pathologies in Romanian population.

**Methods:**

After informed consent, blood samples were collected from 2000 subjects represented by: clinically healthy individuals (*n* = 701) and a group of patients with pathologies linked to low grade inflammation or alteration of carbohydrate metabolism (*n* = 1299). All samples were analysed for the presence of TTV, TTMDV and TTMV DNA by hemi-nested PCR.

**Results:**

The prevalence of TTV, TTMDV and TTMV in the studied population was 68.2, 54.4%, respectively 40.1%, lower than the recent reports from other geographic regions. The three viral species were significantly more frequent in the group of patients compared to the healthy subjects and were associated with type 2 diabetes mellitus. The presence of anelloviral DNA was also associated with medical procedures (e.g. haemodialysis/transfusions, surgical procedures) and previous hepatitis A virus infection. Lifestyle choices related to alcohol consumption, smoking, physical activity and living environment were not associated with differences in distribution of the three viruses.

**Conclusion:**

Further evidence is needed to establish a correlation between infection with human anelloviruses and a pathology or group of pathologies.

## Background

Human anelloviruses, namely *Torque teno virus* (TTV)*, Torque teno mini virus* (TTMV) and *Torque teno midi virus* (TTMDV) species are characterized by their staggering prevalence world-wide, generating conflicting views on their pathogenic potential.

The prevalence of TTV DNA in the blood of the general population reaches to 94–95% by the latest reports (Taiwan 95%, Russia 94%) [1, 2]. Reports of TTMDV and TTMV prevalence are heterogeneous and data regarding prevalence in the blood of healthy individuals are scarce. For TTMDV, the prevalence ranges between 14.5% in Iran [3] and 75% in Japan [4], while the prevalence of TTMV varies between 29% in Korea [5] and 84.8% in Japan [4].

Infection with TTV has been tested in order to link it with a wide range of human pathologies (e.g. hepatitis, cancer, haematological and autoimmune disorders - reviewed in [6]). In some studies only some subtypes of torque teno viruses were reported to be associated with human pathology were reported (e.g. TTMDV and TTMV were associated with respiratory diseases [4] and periodontitis [7]). Although the pathological mechanisms of anelloviruses are not fully understood, the latest studies have pointed out several mechanisms by which these viruses could evade and shape the immune response (activation of pro-inflammatory cytokine production TLR-9 [8], rendering infected lymphocytes resistant to immunomodulation by interferon [9]), thus leading to the worsening of pre-existent conditions. Nevertheless, human anelloviruses seem to be acquired early in childhood [4] being associated with respiratory diseases in children [10, 11].

The altered glucose metabolism and modified expression of interleukins were associated with developing a low grade chronic inflammation. Chronic inflammation appears to be a pathophysiological link between a number of common human diseases such as obesity, type 2 diabetes mellitus (T2DM), chronic periodontitis, hypertension and breast cancer [12–14]. Moreover, chronic periodontitis was strongly linked to obesity [15] and diabetes, being previously considered “the sixth complication of diabetes” [16]. Although the association of periodontitis with obesity is not well understood, it is believed that both are related to high levels of pro-inflammatory molecules (e.g. in the gingival crevicular fluids [17, 18]).

Whilst many studies assessed the association between anelloviruses and inflammation, as far as we are aware, there are few studies aiming to identify an association between the presence of human anelloviruses and breast cancer, as well as diabetes and its comorbidities (i.e. hypertension, obesity, and chronic periodontitis). Also, no studies have been conducted to evaluate aspects of lifestyle choices (e.g. smoking, alcohol consumption, physical activity) as potential factors involved in the epidemiology of anelloviral infections. Also, as far as we know, the human anelloviruses prevalence evaluation has not been attempted at a high scale in Romania before.

The aim of this study was to estimate the prevalence of anelloviral DNA in the Romanian human population and to establish an association with a group of pathologies which can exhibit chronic inflammation and/or alteration of carbohydrate metabolism.

## Methods

A total number of 2000 subjects (701 clinically healthy individuals and 1299 patients with pathologies linked to a low grade inflammation or alteration of carbohydrate metabolism; male: female sex ratio 0.80; average age 45 ± 15.2 years) were selected and described in Table 1.

**Table 1:** Subjects’ clinical and paraclinical characteristics

Variable	Healthy (*n* = 701)	Patients (*n* = 1299)	Statistical significance of differences (*p*-value)
Average age (years) ± SD	37.5 ± 11	49.1 ± 12.5	< 0.001^a^
Male/female gender	282/419 (40.2%/59.8%)	612/687 (47.1%/52.9%)	0.003^b^
Smoking^e^	190 (27.1%)	413 (31.8%)	< 0.001^c^
Drinking^e^	128 (18.3%)	192 (14.8%)	0.95^c^
Physical activity^e^	501 (71.5%)	664 (51.1%)	< 0.001^c^
Orthopaedic trauma			
closed bone fractures	103 (14.7%)	328 (25.3%)	NA
open bone fractures	0 (0%)	29 (2.2%)	NA
BMI	23.3 ± 1.8	39.9 ± 3.3	< 0.001^a^
Obesity	0 (0%)	125 (9.6%)	NA
Chronic periodontitis	0 (0%)	413 (31.8%)	NA
T1DM	0 (0%)	161 (12.4%)	NA
T2DM			
overall T2DM patients	0 (0%)	590 (45.4%)	NA
of which undergone hemodyalisis	0 (0%)	150 (25.4%)	NA
Haemodialysis/transfusions	6 (0.9%)	184 (4.2%)	< 0.001^d^
Breast cancer	0 (0%)	103 (7.9%)	NA
History of hepatitis A virus infection	108 (15.4%)	282 (21.7%)	0.001^b^
TTV	410 (58.5%)	953 (73.4%)	< 0.001^b^
TTMDV	325 (46.4%)	762 (58.7%)	< 0.001^b^
TTMV	225 (32.1%)	576 (44.3%)	< 0.001^b^

*T1DM* – type 1 diabetes mellitus, *T2DM* – type 2 diabetes mellitus, *NA* – not applicable ^e^smokers, drinkers and occasional smokers and drinkers; occasional and regular physical activity (as reported by subjects); statistical tests: ^a^independent samples T test; ^b^Chi squared test; ^c^Kruskal-Wallis test; ^d^Fisher’s exact test

All participants gave their informed consent according to the Helsinki Declaration on ethical principles for medical research involving human subjects.

Clinical and paraclinical data and blood samples were collected from all subjects. Subjects were asked to estimate the frequency of physical exercise (no exercise, occasional exercise and regular exercise), smoking and alcohol consumption habits (non-smoker, former smoker, occasional smoker and smoker, respectively, non-drinker, former drinker, occasional drinker and drinker), number of births and surgical procedures.

Genomic DNA was isolated and purified according to the manufacturer’s recommendations from 250 μL of whole blood using a commercial kit (Wizard^®^ Genomic DNA Purification Kit, Promega, WI, USA). TTV, TTMV and TTMDV DNA was identified using a hemi-nested PCR protocol described by Ninomiya et al. (2008) which amplifies a conserved sequence in the 5' UTR of the viral genome.

The first reaction of the hemi-nested PCR was carried out with GoTaq^®^ Green Master Mix (Promega, WI, USA) in a total volume of 15 μL containing: 1X PCR buffer solution, 1.5 mM MgCl_2_, 0.2 mM dNTP mix, 0.5 μM of each primer (NG779, NG780, NG781 and NG782), 0.6 units of GoTaq^®^ DNA-polymerase and 1.5 μL of genomic DNA. The second round of PCR consisted of three individual reactions using sets of primers specific for TTV (NG779, NG780 and NG785), TTMDV (NG795 and NG796) and, respectively TTMV (NG791, NG792, NG793 and NG794). One μL of PCR product from the first reaction was used as a template. The amplification programs and primer sequences were described by Ninomiya et al. (2008).

The amplification products of the second PCR reactions were resolved by agarose gel electrophoresis (2% *w*/*v*, 5 V/cm for 30 min) and visualized under UV light after ethidium bromide staining.

Negative and positive controls were used to validate PCRs. Samples were considered positive when an expected-size amplicon was generated.

Chi squared and Fisher exact test were used to examine possible differences in the incidence of infection among groups of subjects, while independent samples T-test and Kruskal-Wallis test were used to compare means. Odds Ratio was calculated using SPSS Statistics software v 20.0.0 (IBM). Bonferroni corrected alpha of 0.05 / 150 or ∼0.0003 was considered statistically significant. R version 3.3.3 [19] with the “ggplot2” package [20] was used to generate graphics.

MDR (Multifactor Dimensionality Reduction) software (v.2.0.beta 8.4) was used to perform the investigation of statistical epistasis between investigated variables. Best models implying combination of variables were considered based on 10-fold cross-validation and maximum testing accuracy.

## Results

This study revealed the fact that the DNA of torque teno viruses was present in 71.5% of blood samples investigated, the prevalence of TTV, TTMDV and TTMV being 68.2, 54.4%, respectively 40.1%.

**Fig. 1:**
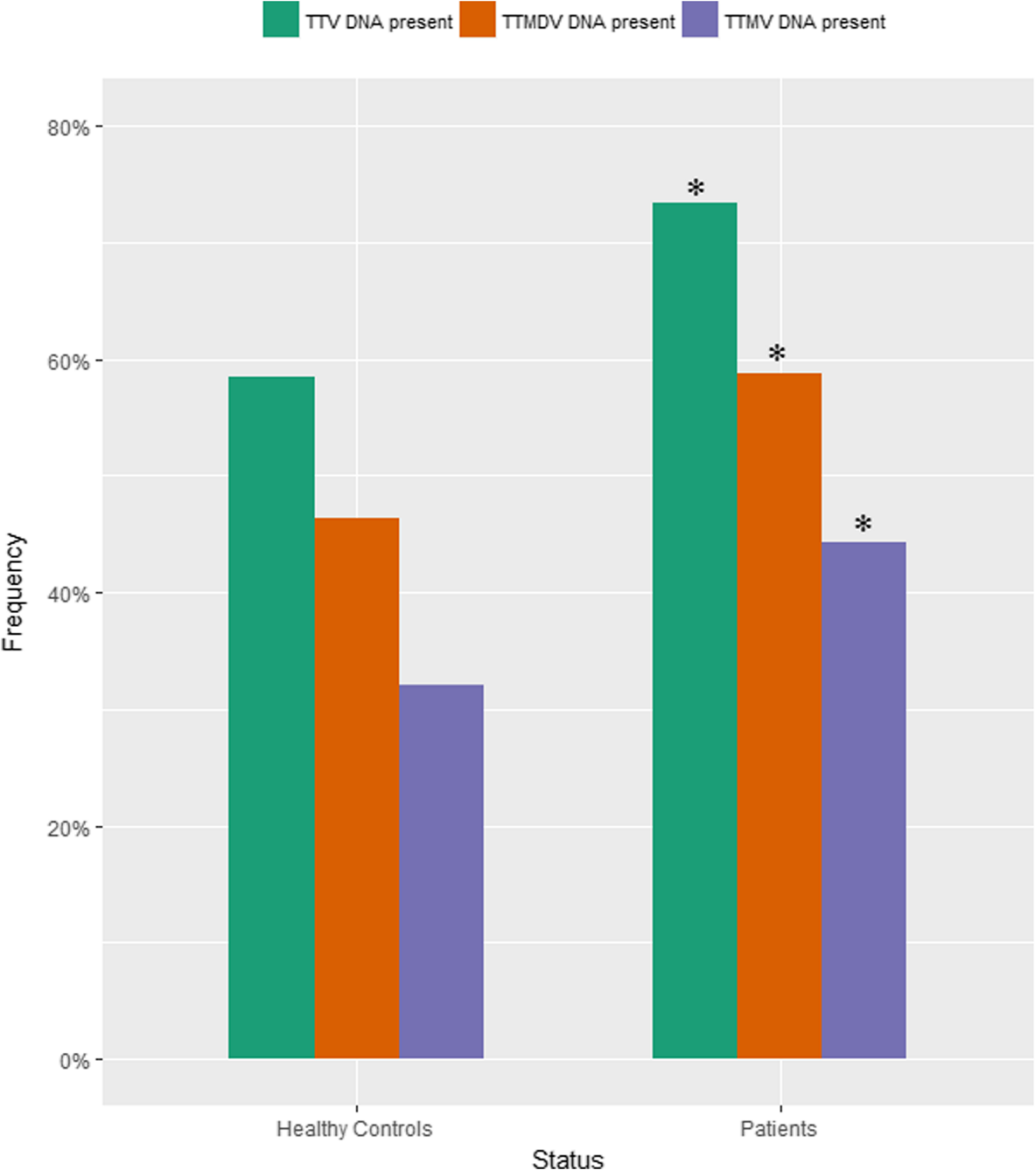
Prevalence of TTV, TTMDV and TTMV DNA in the study group

The distribution of all three viral species considered individually was significantly higher in patients compared to healthy individuals (*p* <  0.0003, Fig. 1).

No difference was noticed in the distribution of the three viruses in men and women participating in this study.

All three anelloviruses were similarly distributed in subjects stratified according to living areas, routine physical activity or alcohol consumption status, both in patients and in controls or also in subjects stratified according to gender.

The risk of achieving an anelloviral infection increases up to 2-fold (OR_TTV_ = 1.8, 95% CI: 1.5–2.3; OR_TTMDV_ = 2, 95% CI: 1.7–2.5; OR_TTMV_ = 1.8, 95% CI: 1.5–2.2) by undergoing at least one surgery and it was higher among men in this group. Of subjects positive for TTV, TTMDV and TTMV DNA, a proportion of 76, 65.2% and, respectively, 47.8%, have undergone at least one surgical procedure (*p* <  0.0003, Table 2).

**Table 2:** Association between the presence of anelloviral DNA and history of HAV infection or different medical procedures in the overall study group

Variable	History of HAV infection (*n* = 390)		Surgical procedures^a^ (*n* = 777)		Undergone dialysis/transfusions (*n* = 190)	
TTV DNA positive	304 (77.9%)* *OR = 1.8 (95% CI 1.4–2.4)*		591 (76%)* *OR = 1.8 (95% CI 1.5–2.3)*		169 (88.9%)* *OR = 4.1 (95% CI 2.6–6.6)*	
	*Men*	*Women*	*Men*	*Women*	*Men*	*Women*
	152/189 (80.4%)* *OR = 2.1 (95% CI 1.4–3.1)*	152/201 (75.6%)**	112/130 (86.1%)* *OR = 3.2 (95% CI 1.9–5.3)*	475/647 (73.4%)* *OR = 2* *(95% CI 1.6–2.6)*	69/79 (87.3%)* *OR = 3.3 (95% CI 1.7–6.6)*	100/111 (90.1%)* *OR = 4.9 (95% CI 2.6–9.3)*
TTMDV DNA positive	260 (66.7%)* *OR = 1.9 (95% CI 1.5–2.4)*		507 (65.2%)* *OR = 2 (95% CI 1.7–2.5)*		149 (78.4%)* *OR = 3.4 (95% CI 2.3–4.8)*	
	*Men*	*Women*	*Men*	*Women*	*Men*	*Women*
	128/189 (67.7%)* *OR = 2.4 (95% CI 1.7–3.3)*	132/201 (65.6%) **	92/130 (70.7%)* *OR = 2.6 (95% CI 1.7–4)*	414/647 (64%)* *OR = 2 (95% CI 1.5–2.5)*	61/79 (77.2%)* *OR = 3.6 (95% CI 2.1–6.2)*	88/111 (79.2%)* *OR = 3.2 (95% CI 2–5.1)*
TTMV DNA positive	220 (56.4%)* *OR = 2.3 (95% CI 1.8–2.8)*		383 (47.8%)* *OR = 1.8 (95% CI 1.5–2.2)*		130 (68.4%)* *OR = 3.6 (95% CI 2.6–5)*	
	*Men*	*Women*	*Men*	*Women*	*Men*	*Women*
	117 / 189 (61.9%)* *OR = 3.5 (95% CI 2.5–4.8)*	103/201 (51.2%)**	83/130 (63.8%)* *OR = 3.5 (95% CI 2.3–5.1)*	300/647 (46.3%)* *OR = 1.6 (95% CI 1.2–2)*	61/79 (77.2%)* *OR = 6.8 (95% CI 3.7–11.1)*	69/111 (62.1%)* *OR = 2.5 (95% CI 1.7–3.8)*

*HAV* – hepatitis A virus, *OR* = Odds Ratio, ^a^at least one surgical procedure including breast cancer surgery, tonsillectomy, appendectomy, caesarean section, surgical abortion, septoplasty or aesthetic surgery; Statistical significance: *Chi Squared/Fisher exact *p* < Bonferroni corrected alpha = 0.0003 (compared with subjects without the analysed variable); **Chi Squared/Fisher exact *p* <  0.05

Human anelloviruses DNA was more frequently found in healthy women who have had at least one surgical abortion or caesarean section/vaginal birth (*p* <  0.0003). Also, the average number of such procedures was higher in women positive for TTV, TTMDV or TTMV DNA compared to those negative for such infections (independent samples T-Test *p* <  0.0003).

Higher prevalence of TTV, TTMDV or TTMV in overall patients and particularly in men (OR_TTV_ = 2.1, 95% CI: 1.4–3.1; OR_TTMDV_ = 2.4, 95% CI: 1.7–3.3; OR_TTMV_ = 3.5, 95% CI: 2.5–4.8) was associated with a history of hepatitis A virus (HAV) infection (Table 2). Bivariate analysis performed with MDR also indicated an association between TTMV, history of HAV infection and haemodialysis (Table 3).

**Table 3:** Association between the presence of anelloviral DNA and couples of variables in the overall study group

Group	Virus	Bivariate analysis	Whole dataset statistics		
		Attributes	Χ^2^	*p*-value	OR
Women	any of the three anelloviruses	surgical procedures^a^ + comorbidities^b^	61.08	< 0.0001	2.83
	TTMV	surgical procedures^a^ + comorbidities^b^	48.05	< 0.0001	2.38
	TTMDV	surgical procedures^a^ + comorbidities^b^	61.08	< 0.0001	2.83
	TTV	surgical procedures^a^ + comorbidities^b^	61.08	< 0.0001	2.83
Men	any of the three anelloviruses	T2DM – HAV	34.35	< 0.0001	2.52
	TTMV	haemodialysis – HAV	67.79	< 0.0001	3.34
	TTMDV	surgical procedures^a^ – HAV	32.79	< 0.0001	2.39
	TTV	surgical procedures^a^ + comorbidities^b^	29.18	< 0.0001	2.22
Total	any of the three anelloviruses	surgical procedures^a^ + comorbidities^b^	88.60	< 0.0001	2.57
	TTMV	surgical procedures^a^ – HAV	80.85	< 0.0001	2.40
	TTMDV	surgical procedures^a^ – T1DM	79.23	< 0.0001	2.29
	TTV	surgical procedures^a^ – HAV	78.82	< 0.0001	2.38

^a^at least one surgical procedure; ^b^any combination of two or more pathologies listed in Table 1

Regarding the association of human anelloviruses with pathology, no significant result was found linking the presence of anelloviral DNA with obesity and chronic periodontitis neither compared with controls, nor compared with normal weight subjects, respectively with periodontally healthy subjects. Likewise, there was no association between the BMI (independent samples T-test > 0.05) and the presence or absence of TTMDV, TTMV or TTV DNA.

The prevalence of anelloviral DNA was significantly higher in patients with hypertension compared with controls (TTV – 74.2% vs. 58.4%; TTMDV – 59.6% vs. 46.3%; TTMV – 46.4% vs. 32.1%).

The distributions of TTV, TTMDV and TTMV presented significant differences between T2DM group compared to healthy subjects (OR = 1.8 - to 2.1, *p* <  0.0003) (Table 4).

**Table 4:** Prevalence of torque teno viruses stratified by gender in the study group

Group	Viral DNA positivity rate		
	TTV	TTDMV	TTMV
T1DM	73.3%**	62.7%* *OR = 1.9 (95% CI 1.3–2.7)*	53.4%* *OR = 2.4 (95% CI 1.7–3.4)*
Men	74.7%** (65/87)	62.1%** (54/87)	46%** (40/87)
Women	71.6%** (53/74)	63.5%** (47/74)	62.1% (46/74)* *OR = 3.309 (95% CI 2–5.5)*
T2DM	75%* *OR = 2.1 (95% CI 1.7–2.7)*	61.8%* *OR = 1.8 (95% CI 1.5–2.3)*	50.3%* *OR = 2.1 (95% CI 1.7–2.7)*
Men	78.1%* *OR = 2.4 (95% CI 1.6–3.4)*	58.9%**	50%* *OR = 2.3 (95% CI 1.6–3.2)*
Women	71.8%* *OR = 1.9 (95% CI 1.3–2.6)*	64.9%* *OR = 2 (95% CI 1. 5–2.7)*	50.6%* *OR = 2.1 (95% CI 1.5–2.8)*
Obesity	70.4%	47.2%	31.2%
Men	68% (44/65)	33.8% (22/65)	26.1% (17/65)
Women	73.3% (44/60)	61.6% (37/60)	36.6% (22/60)
Hypertension	74.2%* *OR = 2 (95% CI 1.6–2.6)*	59.6%* *OR = 1.7 (95% CI 1.3–2.1)*	46.4%* *OR = 1.8 (95% CI 1.4–2.3)*
Men	74.8%* *OR = 2 (95% CI 1.4–2.8)*	57.7%**	45.6%* *OR = 1.9 (95% CI 1.3–2.7)*
Women	73.6%* *OR = 2 (95% CI 1.5–2.8)*	61.5%**	47.2%* *OR = 1.8 (95% CI 1.3–2.4)*
Chronic Periodontitis	74.1%**	58.8%**	42.1%
Men	75.8%	56.3%	43.8%
Women	73.1%**	60.4%	41.1%
breast cancer	80.6%* *OR = 3 (95% CI 1.7–5)*	72.8%* *OR = 2.9 (95% CI 1.9–4.9)*	37.8%
overall patients	73.4%* *OR = 1.9 (95% CI 1.6–2.3)*	58.7%* *OR = 1.6 (95% CI 1.3–1.9)*	44.3%* *OR = 1.7 (95% CI 1.4–2)*
Men	73.4%* *OR = 1.8 (95% CI 1.3–2.5)*	54.4%**	41.7%**
Women	73.4%* *OR = 2 (95% CI 1.6–2.6)*	62.4%* *OR = 1.8 (95% CI 1.4–2.3)*	46.7%* *OR = 1.7 (95% CI 1.4–2.3)*
healthy subjects	58.5%	46.4%	32.1%
Men	59.9%	44%	30.5%
Women	57.5%	48%	33.2%

*T1DM* – type 1 diabetes mellitus, *T2DM* – type 2 diabetes mellitus, *OR* – Odds Ratio, *Chi squared/Fisher exact *p* < Bonferroni corrected alpha = 0.0003 compared with healthy subjects; **Chi squared/Fisher exact *p* < 0.05

**Fig. 2:**
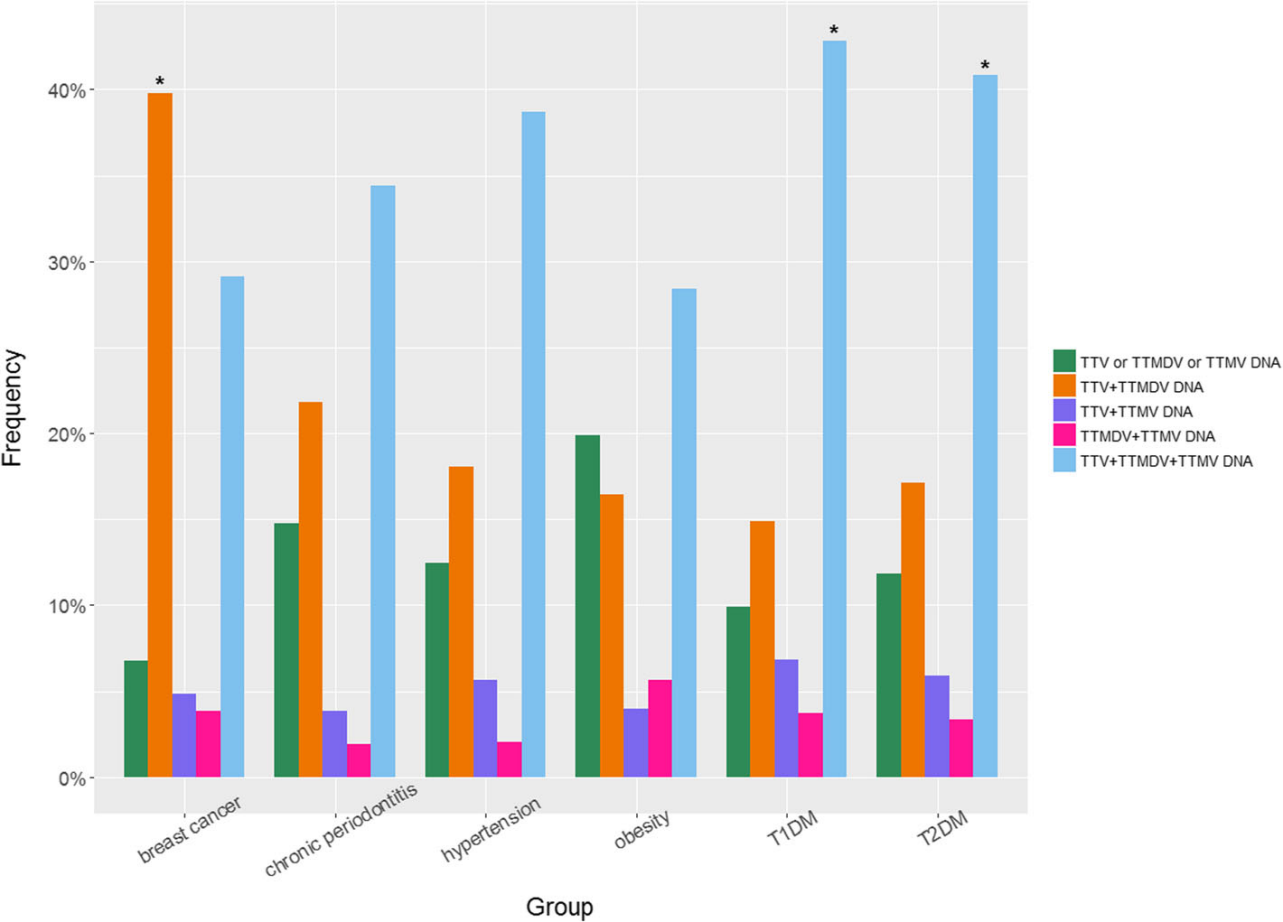
Co-infection pattern distribution of human anelloviruses in overall patients grouped by pathology. *p < Bonferroni corrected alpha = 0.0003

The analysis of co-infection patterns showed that triple co-infection was more frequent in patients with diabetes (both T1DM and T2DM) compared to healthy subjects (*p* <  0.0003). The presence of only TTV and TTMDV DNA was significantly more frequent in patients with breast cancer (*p* <  0.0003) compared with healthy women (Fig. 2).

**Fig. 3:**
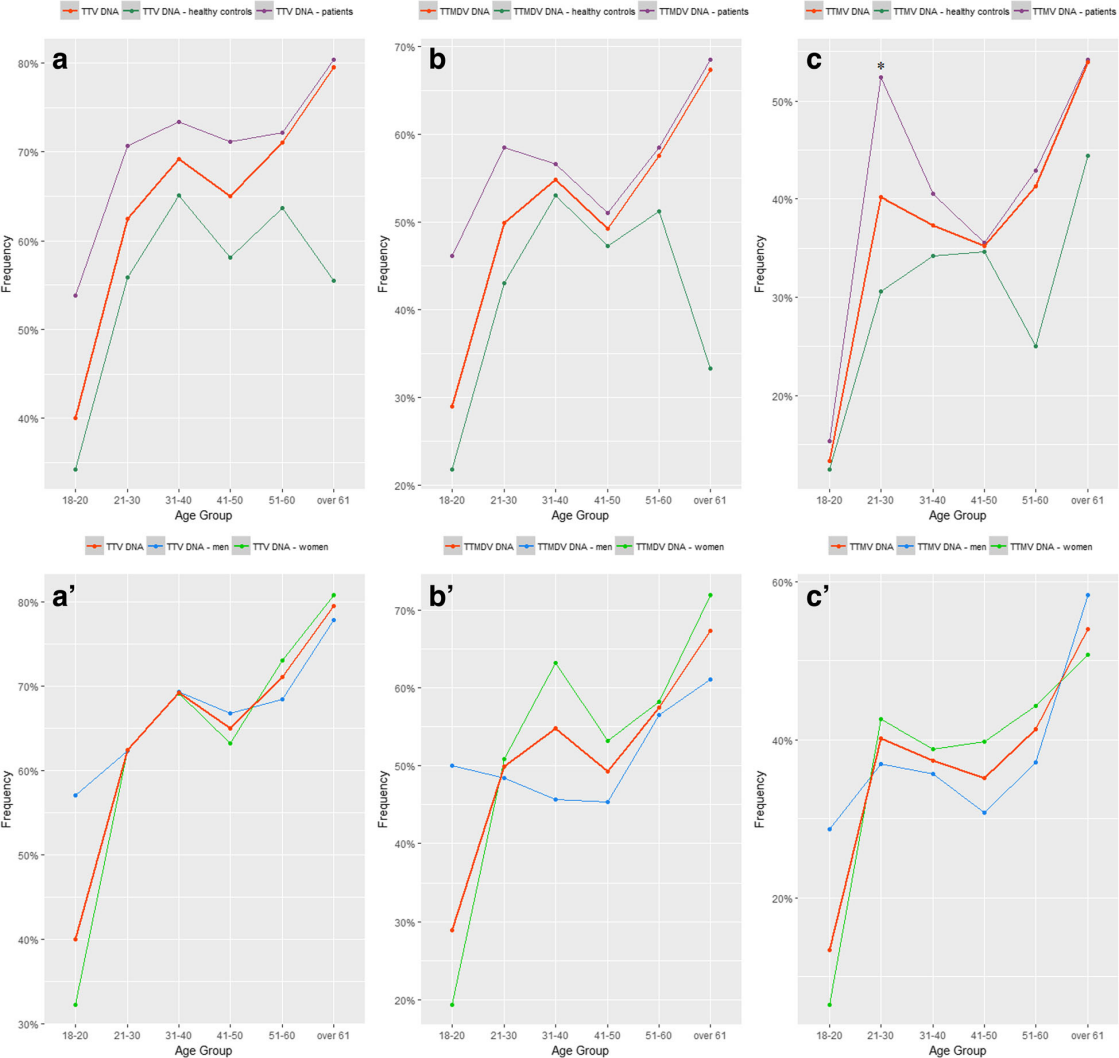
Age group distribution of TTV (**a**, **a’**), TTMDV (**b**, **b’**) and TTMV DNA (**c**, **c’**) stratified by status (top row) and by gender (bottom row) in the overall study group

The analysis of viral DNA distribution among age groups revealed similar patterns for all the three viruses. Human anelloviruses’ prevalence appears to increase with age with a lower point in the 41–50 years group and the highest peak in the over 61 year group (Fig. 3). Nevertheless, there was no significant difference in the frequency of TTV, TTMDV or TTMV DNA between other decades of age.

A significantly higher number of patients within the 21–30 years age group were found positive for TTMV DNA compared to the same age healthy controls (*p* = 0.000031). The significance of this difference is to be further investigated.

The univariate MDR analysis confirmed the obtained results. The bivariate MDR analysis (Table 3) showed that the presence of TTV, TTMDV, TTMV, or at least of one of the viruses is associated with the combination of disease states or particular states (e.g. the presence of at least one anellovirus was associated with the number of surgical procedures and the presence of comorbidities in women).

## Discussion

The prevalence of anelloviral DNA in the overall studied population was 71.5%. Results of this study showed lower values of anelloviruses DNA presence compared to a recent study from Qatar enrolling patients co-infected with HBV and HCV and healthy individuals [21]. Moreover, prevalence values found in the healthy group observed in this study were lower than values reported for only the control group in the study from Qatar (TTV 83.4% vs. 58.5%; TTMDV 74.6% vs. 46.4%; TTMV 62.1% vs. 32.1%). Also, our results are different from those reported for TTV DNA prevalence in healthy individuals of eastern Taiwanese indigenes (95% vs. 58.5%) [1]. Prevalence of viral DNA observed in this study is similar to previous data reported in the saliva of healthy individuals from Romania in 2013 [22]. These results may indicate a possible geographical stratification of anellovirus frequency.

Human anelloviruses have several routes of transmission, including blood [6]. Childbirth or medical procedures that imply open wounds (i.e. various surgical procedures) represent potential gateways for anelloviral particles access into the blood stream; whilst there are no studies to support this theory, in our study significantly higher rates of anelloviral DNA (Table 2) were found in subjects that underwent these procedures. The incidence of TTV DNA in patients undergoing haemodialysis reported by this study (90.6%), all part of the T2DM group, was higher compared to dialyzed patients from Brazil (17–36%) [23] and France (54%) [24]; however the discrepancy can be explained also by the different protocols used for molecular detection of viral DNA. For the patients undergoing haemodialysis (T2DM with chronic renal failure) the significantly higher prevalence of anelloviral DNA can be partially explained by this medical procedure. In addition, another study found that transgenic mice expressing the capsid protein of TTV (encoded by ORF1) showed renal failure resembling nephrotic syndrome [25]. Our data showed TTV, TTVD and TTMV DNA presence in T2DM patients increased up to 4-fold (OR_TTV_ = 4.2, 95% CI: 2.4–7.7; OR_TTMDV_ = 3.6, 95% CI: 2.4–5.4; OR_TTMV_ = 4.1, 95% CI: 2.9–5.9) the association with chronic renal failure compared to T2DM patients negative for viral DNA (*p* <  0.0003); considering these results and previous reports, anellovirus infections may be one of the factors associated with the progression of diabetic nephropathy or with the medical procedures that these patients have undergone.

Evidence showed that TTV has the ability to modulate the response to interferon and to alter the expression of several cytokines [8, 9], thus changing the immune reactivity of the host. Altered expression of pro-inflammatory cytokines predispose to chronic inflammation (which sometimes is represented by persistent, low grade inflammation) which predispose to metabolic dysfunction and may increase the mutation rate [26]. Thus, chronic low grade inflammation was strongly linked to obesity, metabolic syndrome, insulin resistance, hypertension, T2DM [12], chronic periodontitis [17, 18] and some types of breast cancer [14].

TTV DNA was more prevalent among obese women without other comorbidities (Pearson’s Chi squared *p* = 0.024); also BMI of individuals positive for TTV DNA was overall higher than the BMI of TTV DNA-negative individuals (independent sample T-test *p* = 0.03), nevertheless both observations failed to reach statistical significance following Bonferroni correction. These findings could support and come in addition to previous reports of TTV DNA being more frequent with borderline statistical significance (*p* = 0.046) in obese women compared to matched controls [27].

Human anelloviruses exhibit an apparent pan-tropism [6] and its main multiplication cellular component occurs in the T lymphocyte [28]. Findings revealed that the presence of TTV DNA in the gingival tissue is associated with periodontitis [29]. Also, a recent study showed novel species of TTMV associated with periodontitis (*p* = 0.032) [7]. Our results revealed rather the percentage of TTV DNA being higher only in female subjects with periodontitis (p_TTV_ = 0.028). Part of the result may be explained by the fact that women with periodontitis may have several confounding factors [e.g. they have had more surgeries (*p* <  0.001), at least a birth (*p* = 0.005) or haemodialysis or transfusions (*p* = 0.002)]; these attributes were associated with higher prevalence of human anelloviruses in this study. Also, the age of women with periodontitis was higher than periodontally healthy women (average 51.2 vs. 43.5; independent samples T-test *p* <  0.001).

TTV, TTMDV or TTMV DNA was significantly more prevalent in T2DM patients with frequencies of 75, 61.8% respectively 50.3%, while only TTMDV and TTMV DNA was more prevalent in T1DM compared with healthy subjects (Table 4). Anelloviral DNA was more common in patients with hypertension; however, 64.2% of these also had T2DM.

Information regarding the prevalence of human anelloviruses in diabetic patients is limited. Earlier studies reported lower prevalence for TTV DNA in T2DM patients, of 26% (16/60) [30] and 54.7% [24]. Only the second study observed a different distribution of TTV DNA in diabetics vs. control group. Given the complexity of the T2DM group, statistical analysis was performed considering all possible attributes (comorbidities); the prevalence of the three anelloviruses considered individually, as well as triple co-infections were significantly higher in diabetic patients compared to healthy individuals. However, compared with diabetic patients without comorbidities, no significant result was found. This result suggests that comorbidities such as hypertension, obesity or periodontitis do not influence the frequency of human anellovirus infections in T2DM patients. Interestingly, the age of T2DM patients with infections was higher than that of uninfected patients (average age 57.2 vs. 55.7, T-test *p* = 0.004), supporting the observation that incidence of anelloviral infections increases with age.

De Villieres et al. [31] suggested that active infection with TTV promotes a state of chronic inflammation that may contribute as a risk factor for cancer evolution; moreover, the microenvironment of the host cell may impact the viral genome by replication rates or mutation that could lead to the expression of viral proteins with impact on the oncogenic potential. Breast cancer patients from the present study had higher prevalence of TTV and TTMDV DNA compared to healthy women (*p* <  0.0033), as well as the highest rate of TTV and TTMDV co-infection, thus supporting the association between infection with TTV and breast cancer.

The association of different pathologies taken into study with a higher prevalence of TTV, TTMDV, TTMV or different combinations of anelloviruses support the theory that human anelloviruses have different biological behaviour and play distinct roles in pathogenesis and disease, as stated in another study [32].

Further investigation is needed to establish a correlation between prevalence of human anelloviruses and a pathology or group of pathologies.

The distribution of TTV, TTMDV and TTMV DNA in both clinically healthy individuals and patients, men and women, was similarly distributed among age groups and followed almost a tangential curve with the lowest point in the 18–20 age group, an inflection point in the 41–50 year age group and the highest point in the over 61 year age group (Fig. 3). Overall, the number of subjects positive for viral DNA increased with age. This observation comes in support for other studies that reported higher rates of TTV DNA with age [33, 34].

## Conclusions

The prevalence of overall human anelloviruses in the Romanian population was 71.5%, lower than data reported for other regions, thus supporting the geographic stratification of infections with torque teno viruses. TTV was the most prevalent (68.2%) of the three anelloviral species, as reported in most studies.

The distribution of the three viral species was significantly higher in the overall group of patients compared to healthy subjects. The presence of TTV, TTMDV and TTMV was associated with diabetes mellitus and breast cancer.

Current data indicate that torque teno viruses act more likely like an aggravating factor in the evolution of diseases with an inflammatory background, or as an opportunistic agent in people with different medical conditions.

## Abbreviations

HAV: hepatitis A virus
MDR: Multifactor Dimensionality Reduction
OR: Odds Ratio
T1DM: Type 1 Diabetes Mellitus
T2DM: Type 2 Diabetes Mellitus
TTMDV: Torque teno midi virus
TTMV: Torque teno mini virus
TTV: Torque teno virus
